# Predicting delays in service operations

**DOI:** 10.1007/s11628-021-00466-5

**Published:** 2021-10-24

**Authors:** Fabian von Schéele, Darek M. Haftor, Natallia Pashkevich

**Affiliations:** 1 Uppsala University, 75 120 Uppsala, Sweden; 2 Södertörn University, 141 52 Huddinge, Sweden; 3 University of Economics and Human Sciences in Warsaw, Warsaw, Poland

**Keywords:** Service event delay, Service queue, Time delay factor, Delay accumulation, Matrix modelling

## Abstract

Delays constitute a key challenge in the management of service operations, causing substantial quality and cost issues. Delays in one service event can cause delays in another service event and so on, which creates challenges in the management of complex services. Assuming a lower-triangular matrix formalism, we develop a novel approach to modelling such chains of delays in complex service operations such as health care and software development. This approach can enable service managers to identify, understand, predict and control delays. Our research provides a novel theoretical contribution to the literature on service delays.

## Introduction

The spread of COVID-19 has posed a major challenge to health care service providers, which typically lack the capacity to manage such high volumes of incoming patients (Liu et al. 2020; López-Cabarcos et al. 2020). Hospitals have been forced to reorganise their operations to handle these large inflows of severely affected patients. Accordingly, service operations in areas such as oncology and surgery have been reallocated to care for COVID-19 patients (Wong et al. 2020). As an unintended consequence, these resource reallocations have caused major delays in the provision of many regular health care services such as cancer screening (Hamilton 2020; Maringe et al. 2020), planned surgery (Søreide et al. 2020), orthopaedic services (Khan et al. 2021) and cardiology (Aldujeli et al. 2020). Seemingly unrelated health care service systems have affected each other’s capacity to deliver services in a timely fashion because coping with COVID-19 has required additional resources (Sud et al. 2020). These reallocations of service resources have generated chains of delays in the production and delivery of many planned health services (Scala et al. 2020; Søreide et al. 2020), resulting in patient queues that pose a challenge to the management of health services and ultimately threaten people’s safety and well-being (Ageta et al. 2020; Kumar and Dey 2020; Reinstadler et al. 2020). The issues associated with the accumulation and relocation of temporal delays are not unique to health care services. They emerge in all kinds of non-trivial service operations (Oliva 2001; Furrer et al. 2020), including software programming (Choetkiertikul et al. 2015), legal advisory services (Bektas et al. 2021) and manufacturing (Brax 2005).

Recent research has shown that *service time* is a key challenge to service quality, profitability and productivity (von Schéele et al. 2019). The literature addresses various kinds of temporal challenges to service operations (Furrer et al. 2020) yet ignores temporal accumulations of service delays *within* a given service system and the subsequent relocation of these delays *across* several interacting service systems. In such situations, delays tend to accumulate and influence each other, such that a delay in one service event causes delays in another event. These delays can in turn cause delays in other service events, and so on. These cumulative effects of delays within and across service systems are particularly difficult to manage and resolve, hence the focus of the present paper. To close this knowledge gap, we address two research questions: first, *how can we formally estimate the accumulation of temporal delays in service operations*, and consequently, *how can we predict the timing and volume of delays in service operations?* The aim of this study is to provide service managers with tools to understand and predict the emergence of service delays, control them and then increase the quality and productivity of service operations.

Methodologically, we use a conceptual approach to develop formal models that account for delays and their accumulation in service operations. We develop a novel formalism based on lower-triangular matrix modelling. This approach is particularly suited to understanding how a large number of service events can generate delays and influence delays in other services. The proposed formalism accounts for the empirical fact that delays caused in one service event cause delays in another service event, thereby leading to the accumulation of delays in a large chain of events that are difficult to understand, predict, control and therefore manage. Along with the development of this formalism, we present several numerical examples to illustrate the mechanics involved.

The paper continues with a review of the related literature and then defines the key notions of time, delays and time measurement. The notion of using a service matrix as a way of formally modelling a set of interrelated service events is then introduced. This approach enables us to formalise time delays and articulate a novel time delay factor.

Overall, this paper contributes by providing a novel formalisation of temporal delays for a set of service events. For the first time, we model the cumulative effects of delays in a chain of service events. This contribution has specific practical implications for managers who must deal with large temporal delays in downstream service events, such as health care services during the COVID-19 pandemic.

## Literature

Several conceptions of what a *service* is and is not have been proposed in an attempt to unify these ideas (e.g. Wang et al. 2014). For the purposes of the present paper, we adopt a recent conception of a service based not only on operations and economics but also on legal and contractual aspects. Two key merits of this notion are that it accounts for all kinds of services that we experience and that it offers a precise distinction between a service and a good. As explained by Kayastha (2011, p. 319), ‘service exchanges are those contracts in which contracting parties fulfil their all contractual obligations *over a period of time*’, whereas ‘non-service exchanges are those contracts in which contracting parties fulfil their all contractual obligations *in zero time-periods*’ (italics added). These definitions use the notion of *time* to distinguish between a service and a good, whereas a service exchange requires a period of time, an exchange involving goods does not. Accordingly, time is a crucial dimension of a service. This idea has recently received attention in broader conceptions of management and organisation studies (Feldman et al. 2020; Shipp and Jansen 2021), sociology (Rosa 2013; Hassard 2016) and philosophy (Hägglund 2019). At least two temporal aspects are crucial for a service. One is the total required time for the production of a service, and the other is the timing of its delivery (von Schéele et al. 2019). Both aspects condition the costs of a service and the perceived quality of service delivery, and both may influence the potential revenue generated by a given service (Zeithaml et al. 2018). Temporal delays typically exert a negative impact on both the duration of service production and the timing of service delivery (von Schéele et al. 2019). This impact conditions a service’s costs, perceived quality, revenue potential and therefore profit. Temporal delays in service production and delivery form the focus of the present paper.

Temporal delays are a key challenge in nearly all kinds of service provision (Wang and Zhou 2018), from low-skilled restaurants and cleaning services (Kim et al. 2018; Tan and Netessine 2020) to sophisticated health care, legal advisory services and complex software projects (Choetkiertikul et al. 2015). To deal with delays in service operations, practitioners take various approaches. One is to delay planned service delivery. Another is to reduce the content or quality of service delivery. A third is to acquire additional resources to increase operational capacity (Afolalu et al. 2019). The latter approach becomes a key challenge when capacity is acquired from other related service operations because doing so tends to produce delays there (Maglio and Spohrer 2008). This frequent accumulation and relocation of delays between various service events is the focus of the current study (Oliva 2001; Prange 2021).

Although delays in services have been researched for many years, there are several knowledge gaps in relation to the complexity of time in service operations. One research stream focuses on estimating the service event execution capacity required to avoid service delivery delays, which is essentially a question of capacity allocation and the prediction of this capacity allocation (e.g. Banerjee and Gupta 2012; Claeys et al. 2013; Baetens et al. 2018). A second stream of research focuses on the fact that service customers who experience service delivery delays may be affected by those delays in such a way that the time needed to consume a subsequently received service increases. This increase in turn increases the required service production capacity and reduces the perceived quality of the delivered service (Kc and Terwiesch 2009; Kim et al. 2015; Chan et al. 2017). A third research stream offers a new set of service delay predictions, including those based on service history and service content, to detect and predict the emergence of delays (Ibrahim and Whitt 2011; Akşin et al. 2013; Ibrahim 2018). A fourth stream is focused on investigating the distinction between service queues for a specific service versus service queues when several kinds of services are shared. Research in this area has shown that dedicated service queues produce shorter waiting times than shared services (Song et al. 2015; Shunko et al. 2018; Wang and Zhou 2018). The common approach in these research streams is to predict forthcoming variations in service demands and thereby ensure service operation capacity (Afolalu et al. 2019). However, a second approach is to establish flexible service capacity strategies that can deal with unpredicted variations in service demand (Moeller 2010). One solution is to reallocate resources from one service event to another (Ostrom et al. 2015). Whilst this reallocation may solve the problem of meeting demand in the service event that receives additional capacity, it tends to cause a lack of capacity in the other event, thereby relocating a delay from one service event to another. Although all these research contributions confirm the importance of time in service production and delivery, they do not address the fact that delays in the execution of service events tend to be accumulated and relocated (Senderovich et al. 2015). That is, these research contributions fail to consider the fact that delays in one service event cause delays in another subsequent or parallel service event, which then cause delays in other services, and so on (Butcher and Kayani 2008). Thus, large temporal volumes of delays can accumulate across various interconnected service operations, becoming difficult and costly to manage (Chan et al. 2017; von Schéele et al. 2019). This issue is addressed by this paper, which offers a novel way to model the accumulation of delays and the reallocation of resources in services.

## Time, delays and time measurement

The notion of clock time corresponds to the notion of physical time. This form of time is defined in relation to a particular physical event, where a clock second corresponds to the duration of a specific number of periods of radiation of the caesium atom in its ground state at a temperature of 0 K (ISU 1998). More generally, clock time is understood here as the socially accepted notion of what a clock measures in terms of hours, minutes and seconds (Butler 1995). The universality of this agreement is at the heart of civilisation, affecting areas as diverse as contracts specifying employment and product delivery, taxation, corporate and government budgets, medical treatments, school teaching and the legitimacy of government mandates.

For a given service event (action, activity, process or project), there is a temporal start and end. The management of services involves some kind of resource planning and budgeting, including a plan for how much time a service event should consume. A temporal discrepancy may then be defined as the difference between the planned time to conduct a service event and the actual time used to conduct that event. Thus, duration can be understood as end time minus start time.

A peculiar characteristic of human experience of time is irreversibility, which implies that work cannot be recaptured and that a delay cannot be recovered in absolute terms as the passed time has passed forever. The notion of the irreversibility of the constant flow of time leads to a distinction between the production and delivery of services and goods. Resources deployed for the production of services must consume time, whereas goods can typically be stored for later delivery. Because of this irreversibility of time, a delayed work task that is supposed to be conducted in a given time frame, say week *i*, may actually be conducted in week *i* + 1. Accordingly, a temporal queue forms because planned (or budgeted) work for week *i* is postponed to week *i* + 1. These arguments provide us with two fundamental assumptions about time that underlie the model developed in this paper: the irreversibility of time and temporal queues.

## Delays as time discrepancy

Time discrepancy is formalised here as the predefined working time *t*_*b*_, or ‘budgeted time’, as a share of ‘reported time’, *t*_*r*_. It is the relative difference between *t*_*b*_ and *t*_*r*_ for a given service event *j*, as expressed in Eq. 1:

1 $${\tau }_{j}= {\left(\frac{{t}_{r}}{{t}_{b}}\right)}_{j}.$$

In Eq. 1, we assume that ${t}_{r}$ corresponds to the *mean value* of several time records from a set of employees when reported time is compared to budgeted time.

Empirical experience shows that services are prone to delays. Therefore, the relative measure of time distortion (${\tau }_{j}$) typically exceeds 1. Because time distortion refers to a certain time duration of a given service event, information about this duration can also be obtained. Based on Eq. 1, the reported time that *exceeds* the budgeted time for a given service event is defined as a time delay as follows:

2 $$Time\,delay_{j} = d_{j} = \left( {\tau - 1} \right)_{j} .$$

The term ${d}_{j}$ not only expresses a delay of working time but also serves as an implicit measure of an expected temporal queue before a subsequent service event can be conducted. The following example illustrates these fundamental temporal relations.

### Example 1: estimation of time discrepancy

We assume that a period of 120 h is budgeted for the production of a given service (e.g. software code). After completion of that service production, 132 working hours are reported as having been consumed by the software engineer. Time discrepancy is therefore

3 $$\tau = \left(\frac{132}{120}\right)=1.1.$$

The time needed to produce that service has thus been underestimated. Therefore, the mean value of the recorded working hours corresponds to 132 h. Consequently, the time delay caused by this time discrepancy is as follows:

4 $$d= \left(1.1-1\right)=0.1.$$

This value of $d$ can be understood as a time delay of 10% with respect to the budgeted time of 120 h, or more specifically, $0.1\times 120=\rm {delay of} 12 \rm {h}$. Time discrepancy (${\tau }_{j}$) and time delay (${d}_{j}$) form the basis of the model presented in this paper.

## The service matrix

In the following development of the model, we conceive of services in terms of a *service system S*. A given service system is understood as any coherent collection of services constituted by activities with inputs and outputs, actors and resources that conduct those activities, and rules and goals that govern how the activities are conducted (de Véricourt and Perakis 2020). Depending on the interests of the analyst of a given service, the service can be freely denoted as a service or as a set of services. For example, the operation of a conventional restaurant can be regarded as one service system or as several interrelated service systems, including the delivery of food to the restaurant, the preparation of the food, the receipt of orders, service of the food and payment.

To work with this form of interrelated service production in analytical terms and to assess the respective consumption of time, we use a lower-triangular *n* × *n* matrix (Caswell 2001). In such a matrix, which is square with *n* columns and *n* rows, all elements above the main diagonal are equal to zero. Each column represents an interval of working time of a service worker. For illustrative reasons, this interval is assumed to be one working week, which is equivalent to 40 h. This conception of a given service means that each column corresponds to a working week (40 h). Accordingly, column 1 represents week 1, column 2 represents week 2 and so on. Each row represents the total sum of budgeted hours for a week. For example, if the production of a service such as writing software code accounts for 10 service producers (i.e. software engineers) each budgeted to work 40 h per week, then the row corresponds to 400 budgeted working hours in a working week. Using this conceptualisation, we can apply Eqs. 1 and 2 to model the budgeted time for planned service production for the week and then the reported time for the consumed working time that week. We can thus account for the pattern of relationships between budgeted time and reported time for a given service production. Additionally, this matrix lets us account for relationships between several service time frames, which in this case correspond to several weeks of service production. We define the matrix elements in terms of time discrepancy (${\tau }_{j}$) and time delays (${d}_{j}$), where each element represents a relative time discrepancy and time delay with reference to the budgeted working time for the event of 1 week of work (*j*). When referring to a given row or column in the matrix, we use the letter *i* for rows and *j* for columns. When referring to a general column (i.e. week), we write only *j*. In sum, each column of the matrix represents a certain working week, and the number of weeks corresponds to a total period in a given analysis. For example, a study of services that account for 40 weeks is represented by a 40 × 40 matrix.

## Estimation of time delays

In complex service systems with several subsequent work service events where event *j* is conceived in terms of its budgeted and reported time, temporal delays emerge. These temporal delays are interrelated and influence each other because a delay in an earlier service event may lead to a delay in a subsequent service event. A key question here is how to understand and then predict such delays so that they can be suitably managed.

If we consider the above-defined matrix as a lower-triangular matrix composed of time discrepancies (${\tau }_{j}$) and time delays (${d}_{j}$), then its elements represent the relative discrepancies in budgeted time for a given working week. We define a square lower-triangular *n* × *n* matrix ***A***. We also define an *n* × 1 column matrix ***X(t)***. Here, ***X(t)*** represents the sum of budgeted working hours for each week. Mathematically, the product ***AX(t)*** is an *n*x*1* column matrix ***X(t + 1)***, as expressed in Eq. 5 below:

5 $$X\left(t+1\right)=AX\left(t\right).$$

According to Eq. 5, the budgeted working time *X*(*t* + 1) at the end of a period is equal to the budgeted working time *X*(*t*) at the beginning of the period *multiplied by and added to the relative time discrepancies and time delays from previous weeks *(*periods*). The requirement written in italics is solved by matrix ***A***. Equation 5 can be written explicitly as follows:

6 $$\left(\begin{array}{c}\begin{array}{c}Work\, hours\, end\, per\, (t)\\ {X}_{1}\left(t+1)\right.\\ {X}_{2}\left(t+1)\right.\end{array}\\ {X}_{3}\left(t+1)\right.\\ {X}_{4}\left(t+1)\right.\end{array}\right)= {\varvec{A}}\times \left(\begin{array}{c}\begin{array}{c}Work\, hours\, start\, per\, (t)\\ {X}_{1}\left(t\right)\\ {X}_{2}\left(t\right)\end{array}\\ {X}_{3}\left(t\right)\\ {X}_{4}\left(t\right)\end{array}\right).$$

Each column of the matrix ***A*** corresponds to a week (column 1 represents week 1 and so on). The matrix enables us to identify the time discrepancies and time delays originating in each week of the period. ***A*** can be used as a transformation matrix consisting of elements corresponding to the relative time delay of budgeted working time in the service system each week (see Eqs. 1 and 2).

The elements in ***X(t)*** symbolise budgeted working time at time *t*, whereas the elements in ***X(t + 1)*** symbolise budgeted working time at time *t* + 1 (i.e. *after* a certain duration). Both ***X(t)*** and ***X(t + 1)*** are expressed in weeks (where 1 week = 40 h). However, time discrepancy and time delay are relative measures expressed in fractions of a unit. The relative measures capture how much the budgeted working time changes with respect to the end of the time interval. For instance, $\tau$_*3*_*X*_*3*_(*t*) expresses the number of working hours that were actually consumed in week 3.

Typically, the ideal condition for any service system is to achieve a situation where budgeted time equals reported time. Such equality means that the planned time *before* the service duration corresponds to the time reported *after* the service duration. In such a case, the matrix ***A*** corresponds to the identity matrix ***I*** because the budgeted working time ***X(t)*** corresponds exactly to the reported time ***X(t + 1)***. Should such a scenario occur, no temporal queues or delays would be present in the service system. However, empirical experience shows that complex service systems typically have temporal delays (Butcher and Kayani 2008; Chan et al. 2017), implying that equality between budgeted and reported service time is unlikely.

We now express Eq. 5 in greater detail, which results in the following matrix expression:

7 $$\left(\begin{array}{c}{X}_{1}\left(t+1)\right.\\ {X}_{2}\left(t+1)\right.\\ \begin{array}{c}{X}_{3}\left(t+1)\right.\\ {X}_{4}\left(t+1)\right.\end{array}\end{array}\right)=\left(\begin{array}{ccc}{\tau }_{1}& {0}_{2}& \begin{array}{cc}{0}_{3}& {0}_{4}\end{array}\\ {d}_{1}& {\tau }_{2}& \begin{array}{cc}{0}_{3}& {0}_{4}\end{array}\\ \begin{array}{c}{d}_{1}\\ {d}_{1}\end{array}& \begin{array}{c}{d}_{2}\\ {d}_{2}\end{array}& \begin{array}{cc}\begin{array}{c}{\tau }_{3}\\ {d}_{3}\end{array}& \begin{array}{c}{0}_{4}\\ {\tau }_{4}\end{array}\end{array}\end{array}\right)\left(\begin{array}{c}\begin{array}{c}\begin{array}{c}{X}_{1}\left(t\right)\\ {X}_{2}\left(t\right)\end{array}\\ {X}_{3}\left(t\right)\end{array}\\ {X}_{4}\left(t\right)\end{array}\right).$$

Matrix equality is defined as follows:

8 $${X}_{(t+1)}=A{X}_{t},$$

9 $${for X}_{t}= \left(\begin{array}{c}\begin{array}{c}\begin{array}{c}{X}_{1}\left(t\right)\\ {X}_{2}\left(t\right)\end{array}\\ {X}_{3}\left(t\right)\end{array}\\ {X}_{4}\left(t\right)\end{array}\right) \rm {and} A=\left(\begin{array}{ccc}{\tau }_{1}& {0}_{2}& \begin{array}{cc}{0}_{3}& {0}_{4}\end{array}\\ {d}_{1}& {\tau }_{2}& \begin{array}{cc}{0}_{3}& {0}_{4}\end{array}\\ \begin{array}{c}{d}_{1}\\ {d}_{1}\end{array}& \begin{array}{c}{d}_{2}\\ {d}_{2}\end{array}& \begin{array}{cc}\begin{array}{c}{\tau }_{3}\\ {d}_{3}\end{array}& \begin{array}{c}{0}_{4}\\ {\tau }_{4}\end{array}\end{array}\end{array}\right).$$

In Eq. 7, ***X***_***2***_**(t)** shows the budgeted sum of working time for week 2 *before* the week has started. Similarly, in Eq. 7, ***X***_***2***_**(t + 1)** shows the budgeted sum of working time for week 2 *after* the week has passed. Accordingly, the sum of working time has a discrepancy for week 2.

In Eq. 7, row 2 of matrix ***A*** shows that two elements generate discrepancies with respect to the budgeted working time. These elements are the delay from the previous week (*d*_1_) and the time discrepancy of the budgeted working time for week 2 (${\tau }_{2}$). The matrix ***A*** multiplies the budgeted working time for week 2 and adds the delay from the previous week. This operation provides the volume of time that should be worked to complete the work of week 2. The following example illustrates this idea.

### Example 2: Estimation of time delay in a project

We assume that service system *S* consists of 4 weeks and that the time discrepancy is 1.1 in week 1, 1.2 in week 2, 1.2 in week 3 and 1.1 in week 4. Following the definition of Eq. 2, the delay in each week can be written as 0.1, 0.2, 0.2 and 0.1, respectively. In addition, each week in system *S* is budgeted for 40 working hours. We also assume that we have 10 employees who report their time, and we follow how the reported working time develops for each week in relation to the budgeted time. More strategically, we wish to predict the probable working time in week 4. Applying Eq. 7, we obtain the following:

10 $$\left(\begin{array}{c}440\\ 520\\ \begin{array}{c}600\\ 640\end{array}\end{array}\right)=\left(\begin{array}{ccc}1.1& {0}_{2}& \begin{array}{cc}{0}_{3}& {0}_{4}\end{array}\\ 0.1& 1.2& \begin{array}{cc}{0}_{3}& {0}_{4}\end{array}\\ \begin{array}{c}0.1\\ 0.1\end{array}& \begin{array}{c}0.2\\ 0.2\end{array}& \begin{array}{cc}\begin{array}{c}1.2\\ 0.2\end{array}& \begin{array}{c}{0}_{4}\\ 1.1\end{array}\end{array}\end{array}\right)\left(\begin{array}{c}\begin{array}{c}\begin{array}{c}400\\ 400\end{array}\\ 400\end{array}\\ 400\end{array}\right).$$

Equation 7 shows that 640 h must be budgeted for work in week 4 if the project is to be finalised on time. In relation to the originally budgeted time, this budgeted time is an increase of $64/40=1.6$. In other words, the working time is 60% higher than initially budgeted. To clarify, the matrix multiplication above is performed as follows:

11 $$\left(\begin{array}{c}440\\ 520\\ \begin{array}{c}600\\ 640\end{array}\end{array}\right)=\left(\begin{array}{c}\begin{array}{c}\begin{array}{c}1.1\times 400+ 0\times 400+0\times 400+0\times 400\\ 0.1\times 400+1.2\times 400+0\times 400+0\times 400\end{array}\\ 0.1\times 400+ 0.2\times 400+1.2\times 400+0\times 400\end{array}\\ 0.1\times 400+0.2\times 00+0.2\times 400+1.1\times 400\end{array}\right).$$

In this example, it is assumed that no decision or action is taken to decrease the delay in the service system in weeks 1 to 3. The example illustrates the problem of *procrastinated duties*, which becomes critical in week 4. In addition, the example shows how seemingly minor time delays in the initial phases of a service can remain hidden but generate major delays later in the project, creating an overwhelming workload and a crisis at the end of the service.

The total accumulated delay by the end of week (phase) 4 is 140 h. Example 3 shows that this delay can be reduced substantially using our *time delay factor*, proposed in the next section.

## The time delay factor

Equation 7 reveals that time delays (*d*) accumulate from earlier service events (in the previous example, an earlier week) and are transferred to later services events. This delay accumulation may create substantial temporal discrepancies between the initially budgeted service time and the time needed to perform such service events further along the chain. The accumulation of delays from one service event to a subsequent service event can easily generate volumes of delays that may be challenging to handle further along the value chain of service operations, even in situations where the time discrepancy of each individual event is moderate. This accumulated effect of delays is denoted here as the *time delay factor*. It is defined as the addition of all earlier delays of the service working time, more formally expressed in Eq. 12 as follows:

12 $$Time\, {Delay\, factor}_{j}={F}_{j}=\sum_{j=1}^{j}{d}_{j-1}{X(t)}_{j-1}.$$

The time delay factor formalises how all former time delays influence the budgeted working time in later events (weeks), thereby creating temporal delays and queues. The time delay factor is the sum of all temporal delays from earlier weeks. This phenomenon is illustrated in row 4 of matrix ***A*** in Eq. 7.

## Management intervention to handle service delays

The operation of service systems often causes temporal delays (Chan et al. 2017; Wang and Zhou 2018) and thereby temporal queues. Thus, it is important for managers to detect and deal with such delays. Assuming that the aim is to deliver a service (e.g. software development) and that this service has a temporal delay, there are two conventional approaches to handling such delays. One is to use the current service workers (e.g. software engineers) to allocate additional working time (i.e. *overtime* for existing workers). The other is to add external workers to produce the focal service (i.e. *outsourcing*). The latter approach implies that managers must recruit external staff from other service systems.

In our model, any management intervention in a service system *S* requires temporal adjustments to the matrix *A*. These temporal adjustments enable us to preserve the predictability of Eq. 7. In the following discussion, we present a modelling technique that aims to account for such managerial interventions and thereby adjust matrix *A* with respect to (i) adapting the level of time discrepancy, (ii) eliminating the temporal delay (factor ${F}_{j}$) and (iii) changing the budgeted working time *X*(*t*)_*j*_ (here, each week of work) through overtime and outsourcing. This approach produces an updated, or corrected, matrix *A*, denoted *A’*, which also includes the updated time discrepancy and time delay values.

Decisions about future overtime and outsourcing hours are based on historical data (Rogge-Solti and Weske 2013) on the delay factor (${F}_{j}$) from previous work periods (weeks). The budgeted overtime in week *j* can therefore be defined as

13 $${L}_{j}\left(t\right)={F}_{j}\left(t\right)= \sum_{n=1}^{j}{d}_{j-1}{X(t)}_{j-1}.$$

According to Eq. 13, the overtime ${L}_{j}\left(t\right)$ in week *j* must correspond to the delay factor ${F}_{j}\left(t\right)$. When we wish to recalculate the budgeted working time for week *j*, we must include the distorted and budgeted working time for the period, ${{\tau }_{j}X}_{j}\left(t\right)$, *plus* the delay factor in Eq. 12, ${F}_{j}\left(t\right)$, *reduced* by the overtime, ${L}_{j}\left(t\right)$. This calculation gives a new, or adjusted, budgeted working time for week *j* corresponding to

14 $${{X}_{j}}^{^{\prime}}\left(t\right)= {{\tau }_{j}X}_{j}\left(t\right)+ \left({F}_{j}\left(t\right)- {L}_{j}\left(t\right)\right).$$

If we combine Eqs. 13 and 14, we obtain Eq. 15 as follows:

15 $${{X}_{j}}^{^{\prime}}\left(t\right)= {{\tau }_{j}X}_{j}\left(t\right)+ \left(1- fr\right){F}_{j}\left(t\right).$$

The expression $\left(1- fr\right)$ in Eq. 15 models the amount of overtime in the service system. The term $fr$ is a fraction between 0 and 1. When $fr$ equals 1, no working time from the former delay factor is transferred to a new time delay *d’* in the time delay matrix ***A***. In such cases $\left(1- 1\right){F}_{j}\left(t\right)=0$, which means that *d’* in the following period (week) becomes zero. This reasoning is illustrated in the following example.

### Example 3: Estimation of required overtime

We assume that a service system *S* consists of 4 working weeks to develop software and that the time discrepancy in each week is as follows: 1.1, 1.2, 1.2 and 1.1, respectively. Following the definition of Eq. 2, the time delay for each working week is 0.1, 0.2, 0.2 and 0.1, respectively. We also assume that the service system *S* has 10 service workers (i.e. software engineers) and that each worker is budgeted for a 40-h working week. Therefore, there is a total budget of 400 working hours for each week. Given the budget and delays, a key managerial question is, *how much overtime is needed in week 3 to catch up with the expected delays?* After week 2, the manager of service system *S* decides to increase work capacity in week 3 to deal with the time delay. The following calculations provide the answer to this question. We apply the definitions from Eqs. 1, 2 and 7:

16 $$A=\left(\begin{array}{ccc}1.1& {0}_{2}& \begin{array}{cc}{0}_{3}& {0}_{4}\end{array}\\ 0.1& 1.2& \begin{array}{cc}{0}_{3}& {0}_{4}\end{array}\\ \begin{array}{c}0.1\\ 0.1\end{array}& \begin{array}{c}0.2\\ 0.2\end{array}& \begin{array}{cc}\begin{array}{c}1.2\\ 0.2\end{array}& \begin{array}{c}{0}_{4}\\ 1.1\end{array}\end{array}\end{array}\right).$$

The delay factor for week 3 can be calculated accordingly. From Eq. 5, the *time delay* factor is given by

17 $${F}_{j}=\sum_{n=1}^{j}{d}_{j-1}{X(t)}_{j-1},$$

18 $${F}_{3}=\left(0.1\times 400+0.2\times 400\right)=120 h.$$

This calculation produces an answer to the question posed earlier. The employees need to work 120 h of overtime in week 3 to reduce the accumulated time delay. A subsequent question is then, *how many working hours must be budgeted for week 3?* We apply Eq. 14 to answer this second question. We assume that the time delay in this service system is handled fully (100%) with overtime and that no outsourcing is used. The estimation is as follows:

19 $${{X}_{j}}^{^{\prime}}\left(t\right)= {{\tau }_{j}X}_{j}\left(t\right)+ {(1-fr)F}_{j}\left(t\right),$$

20 $${{X}_{3}}^{^{\prime}}\left(t\right)= \rm {1,2}\times 400+(1-0)120=600 h.$$

This calculation provides an answer to the second question, showing that 600 working hours must be budgeted in week 3. This figure is the result of summing the 480 h represented by the ordinary working time and the 120 working hours needed to account for the time delay factor. Consequently, we ask, *what is the numerical value of the new transformation matrix A*′*?* The modified matrix *A* is as follows:

21 $${A}^{^{\prime}}=\left(\begin{array}{ccc}1.1& {0}_{2}& \begin{array}{cc}{0}_{3}& {0}_{4}\end{array}\\ 0.1& 1.2& \begin{array}{cc}{0}_{3}& {0}_{4}\end{array}\\ \begin{array}{c}0\\ 0\end{array}& \begin{array}{c}0\\ 0\end{array}& \begin{array}{cc}\begin{array}{c}1\\ 0\end{array}& \begin{array}{c}{0}_{4}\\ 1.1\end{array}\end{array}\end{array}\right).$$

Finally, we ask, what is the time delay for all 4 weeks after the intervention in week 3? To answer this question, we construct the matrix resulting from Eq. 7. To find the delay for the whole 4-week service project, it is sufficient to focus on week 4.

22 $$\left(\begin{array}{c}440\\ 520\\ \begin{array}{c}400\\ 440\end{array}\end{array}\right)=\left(\begin{array}{ccc}1.1& {0}_{2}& \begin{array}{cc}{0}_{3}& {0}_{4}\end{array}\\ 0.1& 1.2& \begin{array}{cc}{0}_{3}& {0}_{4}\end{array}\\ \begin{array}{c}0\\ 0\end{array}& \begin{array}{c}0\\ 0\end{array}& \begin{array}{cc}\begin{array}{c}1\\ 0\end{array}& \begin{array}{c}{0}_{4}\\ 1.1\end{array}\end{array}\end{array}\right)\left(\begin{array}{c}\begin{array}{c}\begin{array}{c}400\\ 400\end{array}\\ 400\end{array}\\ 400\end{array}\right).$$

The matrix shows that the whole project will be delayed by 40 working hours.

## Discussion and conclusion

This paper addresses the challenge raised by the accumulation and relocation of delays in service production. Such delays tend to accumulate within a given service system and to relocate across several interacting service systems. Therefore, considerable delays may arise (von Schéele and Haftor 2018; von Schéele et al. 2019). This situation occurs in any kind of non-trivial service context, including legal advisory services, restaurants, software engineering, and due to the recent COVID-19 pandemic, many health care systems (Liu et al. 2020; López-Cabarcos et al. 2020). Although research has addressed several temporal aspects of service operations (Ibrahim and Whitt 2011; Kim et al. 2015; Baetens et al. 2018), the question of the accumulation and relocation of delays has been overlooked (Kim et al. 2018; Wang and Zhou 2018; Tan and Netessine 2020). When faced with service delays, managers tend either to decrease service performance or to accumulate and temporal debts, which generates unanticipated delays across related service operations (Prange 2021). To address this gap, we propose a novel formalism to characterise, explain and predict temporal delays in complex service contexts. This approach provides a novel time delay factor, which for a given service event, accounts for earlier delays that influence the service event’s budgeted working time, producing delays and queues. The formalism of the time delay factor proposed here shows how to estimate the accumulation and relocation of delays in service production in a formal manner. Consequently, it shows how to predict the timing and volume of delays in service production*.* Contemporary approaches to service delay management tend to focus on either the prediction of the capacity needed to produce the anticipated volume and quality of services (Afolalu et al. 2019) or customer perceptions of service temporality (Ostrom et al. 2015). The approach described in this paper complements previous research by addressing the conception and management of service delays, their accumulation and their relocation.

From a managerial perspective (Al-Omoush et al. 2020; López-Cabarcos et al. 2020; Xie et al. 2020), the time delay factor offers a unique instrument to help service managers understand and predict the emergence of complex time delays and thereby exercise precise managerial control. This managerial control in turn improves the quality and productivity of service execution. Using this approach, service delays can be predicted and thereby handled based on historical data on service operations rather than informal estimates.

The time delay factor proposed here creates opportunities for further developments in the management of service time. One such development could be the formalisation of eigenvalues and eigenvectors of time delays in services. This development could offer a powerful instrument to balance the execution of each service event in a chain and thus reduce delays. It would allow managers of complex services such as large hospitals and industrial project portfolios to understand the interdependencies between service events both within a single service system and between various service systems. A related development would be to create new simulation models and thereby formally validate our time delay factor, creating a powerful managerial tool in the process. There is also a need to test the formalism prevented here in practical scenarios to provide empirical support. Overall, this paper shows that time delays in complex services can be understood, modelled, predicted and thus managed.
